# Pegylated Interferon-α2a Inhibits Proliferation of Human Liver Cancer Cells *In Vitro* and *In Vivo*

**DOI:** 10.1371/journal.pone.0083195

**Published:** 2013-12-12

**Authors:** Hironori Kusano, Jun Akiba, Sachiko Ogasawara, Sakiko Sanada, Makiko Yasumoto, Masamichi Nakayama, Keiko Ueda, Kosuke Ueda, Takashi Kurita, Keita Todoroki, Yumi Umeno, Osamu Nakashima, Hirohisa Yano

**Affiliations:** 1 Department of Pathology, Kurume University School of Medicine, Kurume, Fukuoka, Japan; 2 Department of Clinical Laboratory Medicine, Kurume University Hospital, Kurume, Fukuoka, Japan; Institut für Pathologie, Greifswald, Germany, Germany

## Abstract

**Purpose:**

We investigated the effects of pegylated interferon-α2a (PEG-IFN-α2a) on the growth of human liver cancer cells.

**Methods:**

The effect of PEG-IFN-α2a on the proliferation of 13 liver cancer cell lines was investigated *in* *vitro*. Cells were cultured with medium containing 0–4,194 ng/mL of PEG-IFN-α2a, and after 1, 2, 3, or 4 days of culture, morphologic observation and growth assay were performed. After hepatocellular carcinoma (HCC) cells (HAK-1B and KIM-1) were transplanted into nude mice, various doses of PEG-IFN-α2a were subcutaneously administered to the mice once a week for 2 weeks, and tumor volume, weight, and histology were examined.

**Results:**

PEG-IFN-α2a inhibited the growth of 8 and 11 cell lines in a time- and dose-dependent manner, respectively, although the 50% growth inhibitory concentrations of 7 measurable cell lines on Day 4 were relatively high and ranged from 253 ng/mL to 4,431 ng/mL. Various levels of apoptosis induction were confirmed in 8 cell lines. PEG-IFN-α2a induced a dose-dependent decrease in tumor volume and weight, and a significant increase of apoptotic cells in the tumor. Subcutaneous administration of clinical dose for chronic hepatitis C (3 μg/kg, 0.06 μg/mouse) was effective and induced about 30-50% reduction in the tumor volume and weight as compared with the control.

**Conclusions:**

Although *in* *vitro* anti-proliferative effects of PEG-IFN-α2a were relatively weak, PEG-IFN-α2a induced strong anti-tumor effects on HCC cells *in* *vivo*. The data suggest potential clinical application of PEG-IFN-α2a for the prevention and treatment of HCC.

## Introduction

Interferons (IFNs) are types of cytokine that are produced by host cells, such as leukocytes, in response to inflammation. Since IFNs possess antiviral activity, antiproliferative activity and various immunoregulatory activities, IFN therapy is used to treat patients with chronic viral hepatitis or certain types of cancer including malignant melanoma, acquired immunodeficiency syndrome-related Kaposi’s sarcoma and some hematopoietic malignancies [1,2]. Lai et al also showed that recombinant IFNα is useful in prolonging survival among patients with inoperable hepatocellular carcinoma (HCC) [3]. In addition, some studies showed IFN therapy might prevent either occurrence or recurrence after initial curative therapy of HCC, such as liver resection and radiofrequency ablation, in patient with chronic viral hepatitis [4–7]. This cancer preventive effect of IFNs is regarded mainly as results of their antiviral effect and the consequent suppression of inflammation, and might be due to their direct antitumor effect against clinically undetectable HCC as well. The detailed mechanism of the antitumor effect of IFNs, however, remains obscure. 

 Pegylated interferon-α2a (PEG-IFN-α2a) and pegylated interferon-α2b (PEG-IFN-α2b), which are used to treat patients with chronic hepatitis C virus (HCV) or B virus (HBV) infection, are modified IFNs that have longer serum half-life in body than non-pegylated forms of IFNs, therefore they can be given to patients only once a week, whereas a standard IFN without pegylation used to be injected up to three to five times a week. This once-a-week injection of pegylated IFNs in combination with daily oral dosing of the nucleoside analogue ribavirin has substantially improved the rate of sustained virological response in patients with chronic HCV infection and got a position as the first line therapy [8,9]. We previously reported that PEG-IFN-α2b which contains 12 kDa polyethylene glycol (PEG) has stronger antitumor effects *in vivo* than non-pegylated IFNs and this result might be indicating that continuous IFNs exposure to cancer cells in body is more effective than continual injection [10]. On the basis of above-described background, we examined the growth inhibitory effects of PEG-IFN-α2a which contains two chains of 20 kDa PEG and has the longest serum half-life among clinically available IFNs on liver cancer cell lines *in vitro* and *in vivo*.

## Methods

### Cell Lines and Cell Culture

This study used 11 HCC cell lines (KIM-1, KYN-1, KYN-2, KYN-3, HAK-1A, HAK-1B, HAK-2, HAK-3, HAK-4, HAK-5, and HAK-6) and 2 human combined hepatocellular and cholangiocarcinoma (CHC) cell lines (KMCH-1 and KMCH-2). These HCC and CHC cell lines were originally established in our laboratory, and each cell line retains the morphological and functional features of the original tumor as described elsewhere [11–20]. Since tumorigenicity is higher in HAK-1B and KIM-1 cells than in the other 11 cell lines that we have, we used these two cell lines for *in vivo* study. 

The cells were grown in Dulbecco’s Modified Eagle Medium (Nissui Seiyaku, Co., Japan) supplemented with 2.5% heat-inactivated (56°C, 30 min) fetal bovine serum (FBS, Bioserum, Victoria, Australia), 100 U/mL penicillin, 100 µg/mL streptomycin (GIBCO BRL/Life Technologies, Inc., Gaithersburg, MD) and 12 mmol/L sodium bicarbonate, in a humidified atmosphere of 5% CO_2_ in air at 37°C. 

### IFN and Reagents

PEG-IFN-α2a (PEGASYS^®^, Chugai Pharmaceutical Co., Ltd., Tokyo, Japan) with the specific activity of 1.4 X 10^7^ IU/mg protein and non-pegylated IFN-α2a (Miltenyi Biotec GmbH, Bergisch Gladbach, Germany) with that of 2.0 X 10^8^ IU/mg protein were used in the study. 

 Anti-bromodeoxyuridine (BrdU) antibody and fluorescein isothiocyanate-conjugated goat anti-mouse immunoglobulin (FITC-GAM) were purchased from Becton Dickinson Immunocytometry Systems USA (San Jose, CA); control normal mouse IgG_1_, from DAKO (Glostrup, Denmark); rat antibody against mouse endothelial cells (anti-CD34, clone MEC14.7), from Serotec Co., UK; and mouse monoclonal antibody against human α-smooth muscle actin (SMA) that cross-reacts with mouse α-SMA (clone 1A4).

### Effects of PEG-IFN-α2a on the Proliferation of HCC and CHC Cell Lines *in vitro*

The effects of PEG-IFN-α2a on the growth of the cultured cells were examined with colorimetry using 3-(4,5-dimethylthiazol-2yl)-2,5-diphenyl tetrazolium bromide (MTT) assay kits (Chemicon, Temecula, CA) as described elsewhere [18,21]. Briefly, the cells (1.5~8 X 10^3^ cells per well) were seeded on 96-well plates (Nunc, Inc, Roskilde, Denmark), cultured for 24 hours, and the culture medium was changed to a new medium with or without PEG-IFN-α2a (0.016, 0.064, 0.256, 1.024, 4.096, 16.4, 65.5, 262, 1,048, or 4,194 ng/mL). After culturing for 24, 48, 72 or 96 hours, the number of viable cells was measured with ImmunoMini NJ-2300 (Nalge Nunc International, Tokyo, Japan) by setting the test wavelength at 570 nm and the reference wavelength at 630 nm. To keep the optical density within linear range, all experiments were performed while the cells were in the logarithmic growth phase. 

### Quantitative analysis of apoptotic cells induced by PEG-IFN-α2a

HAK-1B or KIM-1 cells cultured with medium alone (control), non-pegylated IFN-α2a (10 ng/ml=2,000 IU/ml) or PEG-IFN-α2a (144 ng/ml=2,000 IU/ml) for 72 hours were stained with the Annexin V-EGFP (enhanced green ﬂuorescent protein) Apoptosis Detection Kits (Medical & Biological Laboratories Co., Ltd.) according to the manufacturer’s instructions. After staining, the cells were analyzed using a FACScan (Becton Dickinson Immunocytometry Systems, San Jose, CA), and Annexin V-EGFP-positive apoptotic cell rate was determined. 

### Morphological Observation

For morphological observation under a light microscope, cultured cells were seeded on Lab-Tek tissue culture chamber slides (Nunc, Inc.), cultured with or without PEG-IFN-α2a (262, 1,048 or 4,194 ng/mL) for 72 hours, fixed for 10 min in Carnoy’s solution, and stained with hematoxylin-eosine (HE). 

### Effects of PEG-IFN-α2a on HCC Cell Proliferation in Nude Mice

All animal experiments were approved by the institutional committee for animal experiments in Kurume University School of Medicine (Permit Number: 1334), and conducted according to the Guide for the Care and Use of Laboratory Animals of the National Institute of Health and the Regulations for Animal Experimentation of Kurume University School of Medicine. Mice were killed by cervical dislocation under diethyl ether anesthesia, and all efforts were made to minimize suffering. Cultured HAK-1B or KIM-1 (10^7^ cells/mouse) was subcutaneously (s.c.) injected into the backs of 5-week-old female BALB/c athymic nude mice (Clea Japan, Inc., Osaka, Japan). Five to seven days later when the largest diameter of the tumor, which was measured by using caliper, reached approximately 5~10 mm (Day 0), tumor volume (mm^3^) was calculated in the equation ‘the largest diameter X (the smallest diameter)^2^ X 0.5’, and then the mice were divided into 5 groups (n=8 each). Tumor volume was measured on Day 0, 1, 2, 4, 6, 8, 10, 12, and 14. Mouse body weight was measured on Day 0, 8, and 14. After 2-week treatment, mice were killed on Day 15 and the actual tumor weight was also measured. In experiment 1, the 5 groups of 8 mice received either phosphate-buffered saline (PBS) (Control) or PBS with the different dosages of PEG-IFN-α2a (0.06–60 μg) once a week for 2 consecutive weeks (Day 1 and Day 8). The clinical dose of PEG-IFN-α2a in chronic hepatitis C treatment is about 3 µg/kg and is equivalent to the lowest dose (0.06 µg/mouse=840 IU/mouse) in this experiment. After killing, resected tumors were used for morphological studies (e.g., HE staining and immunohistochemistry) and Enzyme-linked immunosorbent assay (ELISA) analysis. Every mouse received an intraperitoneal injection of 1 mg of BrdU 30 min before killing. In experiment 2, to examine the difference between non-pegylated and pegylated IFNs, 5 groups of 8 mice received either PBS (Control), PBS with 0.0042 or 0.042 µg of IFN-α2a (840 or 8,400 IU, respectively), or PBS with 0.06 or 0.6 µg of PEG-IFN-α2a (840 or 8,400 IU, respectively). In this experiment, tumor weights on Day 15 and numbers of apoptotic cells were compared among the groups. 

### Morphological Examination of the Subcutaneous Tumors of Nude Mice

The number of cells showing the characteristics of apoptosis (e.g., cytoplasmic shrinkage, chromatin condensation, and nuclear fragmentation) was counted in at least three 0.25 mm^2^-areas within an HE-stained specimen, and the average number per area was obtained. The TUNEL technique (ApopTag^®^ Peroxidase *In Situ* apoptosis Detection Kits, CHEMICON International, Inc, CA) was used to detect apoptotic cells, and the average number of TUNEL-positive cells per area was obtained, as described above. The specimens were also immunostained for incorporated BrdU using BrdU Staining Kits (Oncogene Research Products, Boston, MA), and the average number of positive cells per area was obtained as described above. In addition, double-immunostaining was performed with anti-mouse endothelial cell antibody, anti-human α-SMA antibody, Histofine simple stain mouse MAX-PO (Rat) kits (Nichirei, Tokyo, Japan), and HistoMouse™-plus kits to detect artery-like blood vessels as described in our previous report [21,22]. The number of double-immunostaining-positive blood vessels in the tumor was counted on each specimen. Granulation tissue within the tumor were excluded in counting of blood vessels. The size of the counted area was measured by tracing the outline displayed on a computer monitor using Mac SCOPE (MITANI Corp., Chiba, Japan). From the obtained number of vessels per unit area (mm^2^), the group mean was obtained for group comparison.

### Enzyme-linked immunosorbent assay (ELISA)

Portions of the resected xenograft tumors were homogenized in 500 μl of ice-cold Ca^2+^ and Mg^2+^-free PBS containing 100 mg/ml phenymethylsulfonyl ﬂuoride using a pellet pestle. The mixture was centrifuged for 10 min (12,000 g, 4°C), and the supernatant was stored at -20°C until use. After the determination of the amount of the tissue protein in the supernatant using a BCA protein assay reagent (Pierce, Rockford, IL), the amount of basic fibroblast growth factor (bFGF) and IL-8 was measured by using commercially available ELISA kits (R&D Systems, Minneapolis, MN).

### Statistics

Comparisons of estimated tumor volume and colorimetric cell growth were performed using two-factor factorial ANOVA and Student’s *t*-test, respectively. The other data comparisons were performed using the Mann-Whitney U test.

## Results

### Effects of PEG-IFN-α2a on Liver Cancer Cell Proliferation *in vitro*

**Figure 1:**
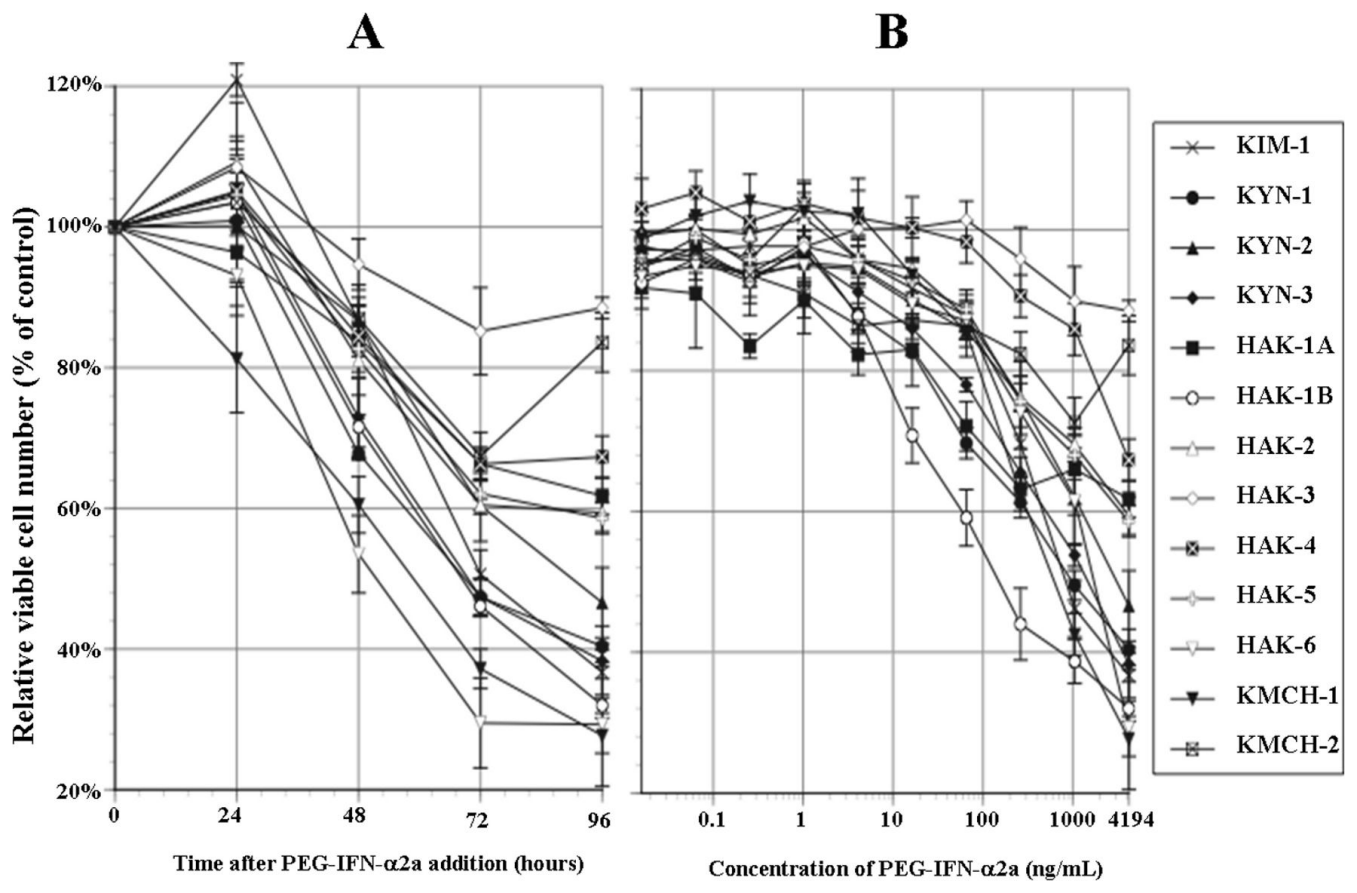
Anti-proliferative effect of PEG-IFN-α2a. (A) Chronological changes in relative viable cell number (% of the control) after adding 4,194 ng/mL of PEG-IFN-α2a. Growth was suppressed with time in 8 cell lines. (B) 96 hours after adding 10 different concentrations of PEG-IFN-α2a. Cell proliferation was suppressed in a dose-dependent manner in 11 cell lines. The suppression was significant (*P* < 0.0001~0.05) in the ranges of 0.016~4,194 ng/mL of PEG-IFN-α2a in HAK-6, 0.256~4,194 ng/mL in KYN-3 and HAK-1A, 4.096~4,194 ng/mL in KIM-1, KYN-1, HAK-1B, HAK-2 and KMCH-2, 16.4~4,194 ng/mL in KYN-2, HAK-5 and KMCH-1, 262~4,194 ng/mL in HAK-4, and at 4,194 ng/mL in HAK-3 (Student *t*-test). Eight samples were used in each experiment (n = 8). The experiment was repeated at least 3 times for each cell line. The figures represent average ± SE of the experiments.

Twenty-four hours after the addition of 4,194 ng/mL of PEG-IFN-α2a, mild increase in the relative viable cell number occurred in 9 cell lines (all cell lines except KYN-2, HAK-1A, HAK-6, and KMCH-1). However, after 72 hours or later, a 10% or more decrease in the cell number occurred in all cell lines (Figure 1A). In HAK-2, HAK-3, and HAK-4, HAK-6, and KMCH-2, proliferation was suppressed up to 72 hours and the cell number reached a plateau or slightly increased thereafter. In the other 8 cell lines, proliferation was suppressed to varying degrees up to 96 hours. 

The relative viable cell number was suppressed in 11 cell lines (all cell lines except HAK-1A and KMCH-2) in a dose-dependent manner after the 96 hours-incubation with PEG-IFN-α2a (Figure 1B). In 7 cell lines (HAK-1B, KMCH-1, KIM-1, KYN-1, HAK-6, KYN-3, and KYN-2), the number was suppressed to 50% or less with 4,194 ng/mL of PEG-IFN-α2a, and the 50% inhibitory concentration (IC50) was 253 ng/mL for HAK-1B, 670 ng/mL for KMCH-1, 1,105 ng/mL for KIM-1, 1,128 ng/mL for KYN-1, 1,302 ng/mL for HAK-6, 1,524 ng/mL for KYN-3, and 4,431 ng/mL for KYN-2. No relationship was detected between the histological differentiation level of the original tumor and sensitivity to the anti-proliferative effect of PEG-IFN-α2a.

**Figure 2:**
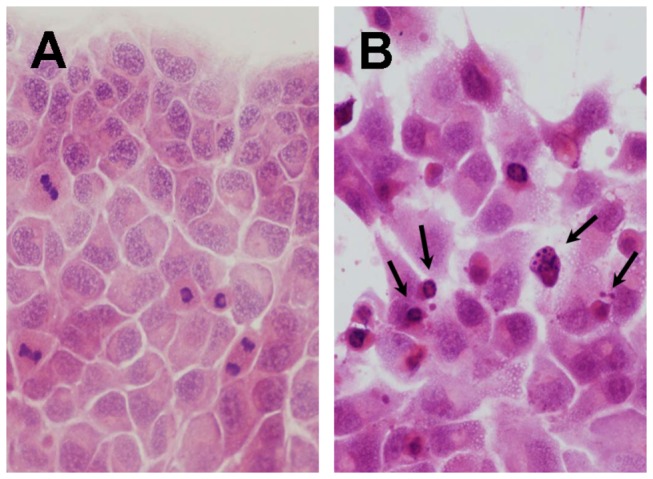
Photomicrograph of HAK-1B cells cultured for 72 hours on a Lab-Tek Chamber slide. (A) Without PEG-IFN-α2a in culture medium. (B) With 4,194 ng/mL of PEG-IFN-α2a in culture medium. Apoptotic cells (short arrows) characterized by cytoplasmic shrinkage, chromatic condensation and nuclear fragmentation were noted (HE staining, X 200).

Seventy-two hours after adding 4,194 ng/mL of PEG-IFN-α2a, 8 cell lines (all cell lines except KYN-3, HAK-1A, HAK-2, HAK-3, and KMCH-2) showed characteristics of apoptosis, e.g., cytoplasmic shrinkage, chromatin condensation, and nuclear fragmentation, in various degrees and in a dose-dependent manner (Figure 2). The appearance of apoptosis was further confirmed in HAK-1B and KIM-1 cells cultured with 10 ng/ml (=2,000 IU/ml) of IFN-α2a or 144 ng/ml (=2,000 IU/ml) of PEG-IFN-α2a by apoptosis detection assay (Table 1). Non-pegylated IFN-α2a induced much more apoptosis than PEG-IFN-α2a. 

**Table 1:** Quantitative analysis of apoptosis in HAK-1B or KIM-1.

	Annexin V-EGFP apoptotic cells (%)		
Cell line^a^	Control	IFN-α2a	PEG-IFN-α2a
HAK-1B	4.1 ± 0.5^b^	18.5 ± 0.3	10.9 ± 0.5
KIM-1	9.4 ± 0.4	47.0 ± 0.2	29.8 ± 2.1

^a^ Cells were cultured with medium alone (Control), IFN-α2a (10 ng/ml=2,000 IU/ml) or PEG-IFN-α2a (144 ng/ml=2,000 IU/ml). ^b^ Mean ±SE.

### Effects of PEG-IFN-α2a on HCC Cell Proliferation in Nude Mice

**Figure 3:**
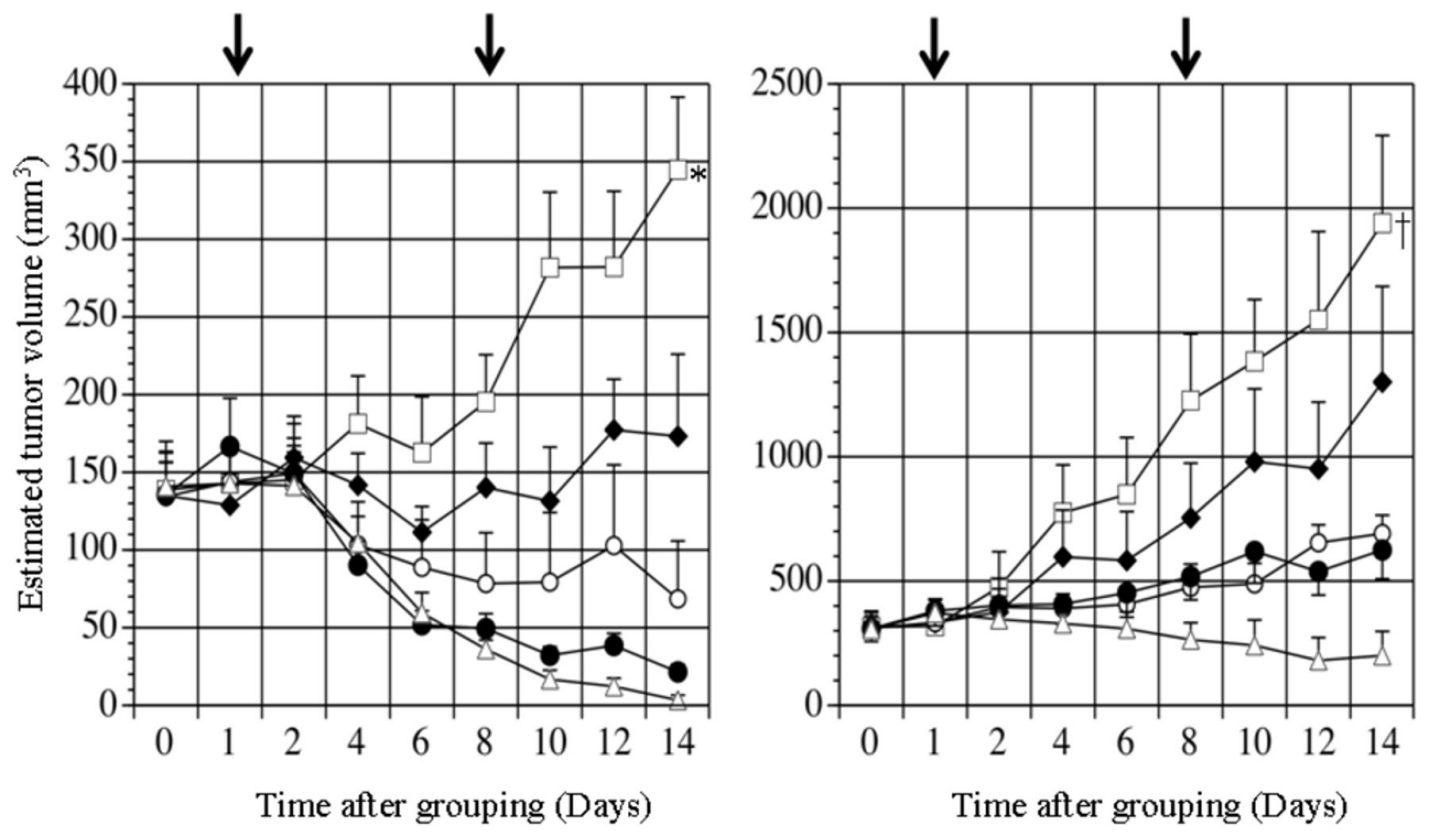
Time-course change in estimated tumor volumes of subcutaneously transplanted HAK-1B (A) or KIM-1 (B) tumors in nude mice in Experiment 1. The mice received a subcutaneous injection of 0.06 (▲), 0.6 (○), 6 (●), or 60 (∆) µg of PEG-IFN-α2a, or medium alone (Control) (□), once a week for 2 consecutive weeks. The arrows show the days of injection. The figures represent average ± SE. *P < 0.0001, versus the other groups. †*P* < 0.01, versus the other groups.

Chronological changes in estimated tumor volume after subcutaneous injection of cultured HAK-1B or KIM-1cells to nude mice are summarized in Figure 3. Dose-dependent suppression of tumor volume was observed in mice receiving PEG-IFN-α2a. In the experiment of HAK-1B tumors, a significant difference in the changes in tumor volume and tumor weight was observed between the Control mice and the mice that received 0.06, 0.6, 6 or 60 μg of PEG-IFN-α2a (*P* < 0.0001 by two-factor factorial ANOVA; and *P* < 0.001~0.02 by the Mann-Whitney U test, Figure 3A and Table 2). In the experiment of KIM-1 tumors, a significant reduction of tumor volume was also observed with the use of PEG-IFN-α2a (*P* < 0.001 by two-factor factorial ANOVA, Figure 3B). There were significant differences in the actual tumor weight between the Control group and the PEG-IFN-α2a groups, except for the PEG-IFN-α2a (0.06 μg) group (Table 2). The actual tumor weight at the end of the experiment 2 was summarized in Table 3. Subcutaneous injection of 0.6 μg of PEG-IFN-α2a induced the significant reduction of tumor weight, compared with the Control group and the group that received the same international unit of non-pegylated IFN-α2a (*P*<0.005 and *P*<0.03, respectively). In this experiment, there was no significant difference between the Control group and the PEG-IFN-α2a (0.06 μg) group (*P*=0.078). 

**Table 2:** The weight of subcutaneous tumors of HAK-1B or KIM-1 cells in nude mice at killing (Experiment 1).

	Tumor weight (g)	
Treatment group^a^	HAK-1B	KIM-1
Control	0.303 ± 0.05^b^, ^c^	1.050 ± 0.24^e^
PEG-IFN-α2a (0.06 μg)	0.141 ± 0.03^d^	0.725 ± 0.17^f^
PEG-IFN-α2a (0.6 μg)	0.033 ± 0.01	0.439 ± 0.04
PEG-IFN-α2a (6 μg)	0.015 ± 0.01	0.434 ± 0.04
PEG-IFN-α2a (60 μg)	0.0	0.076 ± 0.05

^a^ Cultured HAK-1B or KIM-1 cells (1.0 X 10^7^) were subcutaneously transplanted into nude mice. Five groups of 8 mice received either phosphate-buffered saline (PBS) (Control) or PBS with the different dosages of PEG-IFN-α2a (0.06–60 μg) once a week. All mice were killed and the tumor weight was measured on the 15th day. ^b^ Mean ± SE. ^c^ *P* < 0.02, versus the PEG-IFN-α2a (0.06 μg) group; *P* < 0.001, versus the PEG-IFN-α2a (0.6 μg) group; *P* < 0.001, versus the PEG-IFN-α2a (6 μg) group. ^d^. *P* < 0.02, versus PEG-IFN-α2a (60 μg). ^e^ Not significant, versus the PEG-IFN-α2a (0.06 μg) group; *P* < 0.03, versus the PEG-IFN-α2a (0.6 μg) group; *P* < 0.05, versus the PEG-IFN-α2a (6 μg) group; *P* < 0.01, versus the PEG-IFN-α2a (60 μg) group. ^f^. *P* < 0.05, versus the PEG-IFN-α2a (60 μg) group.

**Table 3:** The actual weight and numbers of apoptotic cells of subcutaneous tumors at killing (Experiment 2).

Treatment group^a^	activity of interferon (IU)	Tumor weight (g)	Apoptosis (Number of cells/0.25mm^2^)
Control	0 IU	0.726 ± 0.09^b, c^	7.6 ± 0.9^d^
IFN-α2a (0.0042 µg)	840 IU	0.588 ± 0.07^d^	7.9 ± 0.9^g^
IFN-α2a (0.042 µg)	8,400 IU	0.531 ± 0.04^e^	7.6 ± 0.7^h^
PEG-IFN-α2a (0.06 μg)	840 IU	0.493 ± 0.04^f^	8.9 ± 0.9
PEG-IFN-α2a (0.6 μg)	8,400 IU	0.355 ± 0.03	9.7 ± 1.0*

^a^ Cultured HAK-1B cells (1.0 X 10^7^) were subcutaneously transplanted into nude mice. Five groups of 8 mice received either PBS (Control), PBS with 0.0042 or 0.042 µg of IFN-α2a (840 or 8,400 IU, respectively), or PBS with 0.06 or 0.6 µg of PEG-IFN-α2a (840 or 8,400 IU, respectively). All mice were killed and the tumor weight was measured on the 15th day. The number of apoptotic cells was counted in at least three 0.25 mm^2^-areas in each section stained with hematoxylin and eosin, and the average number per area in each group was obtained. ^b^ Mean ± SE. ^c^ *P* < 0.005, versus the PEG-IFN-α2a (0.6 μg) group. ^d^ *P* < 0.02, versus the PEG-IFN-α2a (0.6 μg) group. ^e^ *P* < 0.03, versus the PEG-IFN-α2a (0.6 μg) group. ^f^ *P* < 0.02, versus the PEG-IFN-α2a (0.6 μg) group. ^g^ *P* < 0.05, versus the PEG-IFN-α2a (0.6 μg) group. ^h^ *P* < 0.001, versus the PEG-IFN-α2a (0.6 μg) group.

**Figure 4:**
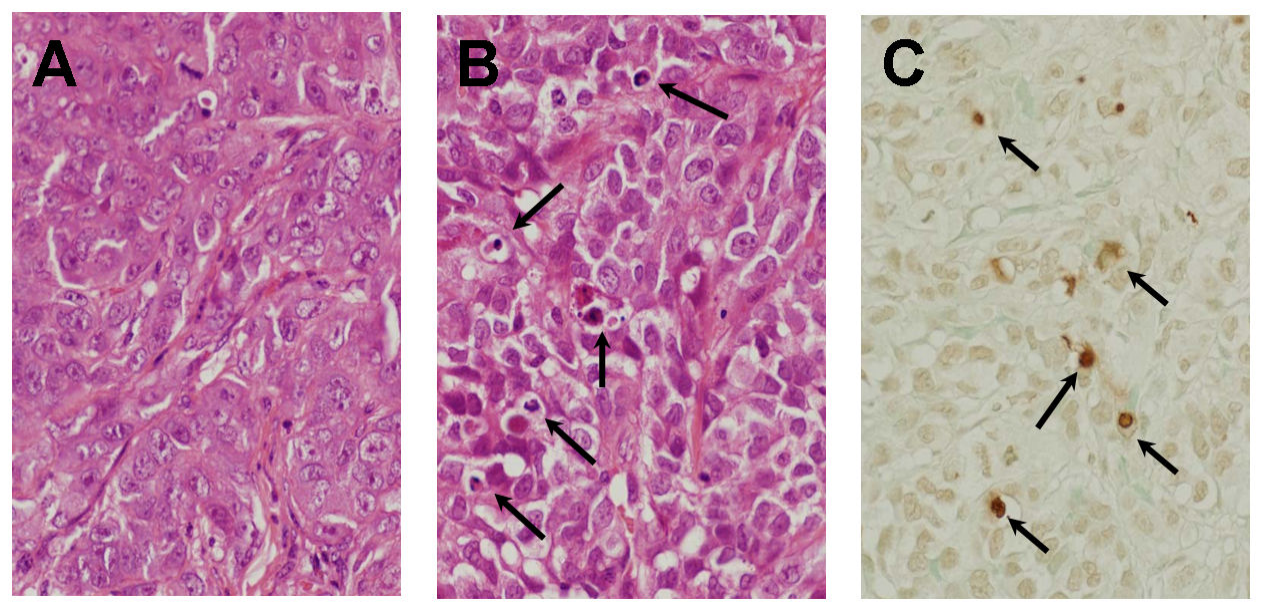
Photomicrograph of subcutaneous human HCC tumor in nude mice that was developed after the injection of HAK-1B cells. (A) A control mouse that received culture medium alone. The tumor shows a compact arrangement of tumor cells and a sinusoid-like structure in the stroma. (B) A mouse that received a s.c. injection of 0.06 μg of PEG-IFN-α2a. There are some apoptotic tumor-cells characterized by shrinkage and eosinophilic change in the cytoplasm, chromatin condensation and/or fragmentation of nuclei (arrows, HE staining, X200). (C) The same tumor as shown in (B). There are some TUNEL-positive cells showing brown nuclei (arrows, stained by the TUNEL technique, X200).

Histological examination of the HAK-1B tumor specimens stained with HE revealed that the numbers of apoptotic cells in the mice treated with PEG-IFN-α2a (0.06 or 0.6 μg) were significantly higher than that of the Control, and the number increased dose dependently (Figure 4, A and B; Table 4). The incidence of apoptosis in TUNEL-stained sections showed the same tendencies as those obtained in HE-stained sections (Figure 4C and Table 4). Immunohistochemical examination of BrdU uptake in HAK-1B tumors revealed that there was no significant difference in BrdU labeling index between the Control and PEG-IFN-α2a (0.06 or 0.6 μg) groups (Table 4). As for apoptosis, similar findings were observed in experiment 2 in which KIM-1 was used. The group treated with 0.6 μg of PEG-IFN-α2a showed increased number of apoptotic cells than the control group. There was no significant difference between the control and IFN-α2a group. In addition, the group treated with 0.6 μg of PEG-IFN-α2a (8,400 IU) showed higher number of apoptotic cells than those with 0.042 µg of IFN-α2a (8,400 IU).

**Table 4:** Numbers of apoptotic cells and BrdU-positive cells in human HCC tumors subcutaneously transplanted in nude mice.

	Apoptosis^b^ (Number of cells/0.25mm^2^)		BrdU Labeling Index^c^ (Number of positive cells/0.25mm^2^)
Treatment group^a^	HE stain	TUNEL method	
Control	8.4 ± 0.8^d,e^	9.6 ± 1.1^e^	32.3 ± 1.6^f^
PEG-IFN-α2a (0.06 μg)	12.2 ± 1.0	15.4 ± 1.8	27.0 ± 2.6
PEG-IFN-α2a (0.6 μg)	12.4 ± 0.9	16.1 ± 1.5	31.3 ± 6.9

^a^ Cultured HAK-1B cells (1.0 X 10^7^) were subcutaneously transplanted into nude mice. Five groups of 8 mice received either phosphate-buffered saline (PBS) (Control) or PBS with the different dosages of PEG-IFN-α2a (0.06–60 μg) once a week. Tumors of mice that received 6 or 60 μg of PEG-IFN-α2a could not be used because the tumors were too small to evaluate. All mice were killed on the 15th day. ^b^ The number of apoptotic cells was counted in at least three 0.25 mm^2^-areas in each section stained with hematoxylin and eosin, and the average number per area in each group was obtained. The number of TUNEL-positive cells was also counted in the same manner. ^c^ The number of BrdU-positive cells was counted in at least three 0.25 mm^2^-areas in each section, and the average number per area in each group was obtained as the labeling index. ^d^ Mean ± SE. ^e^ *P* < 0.02, versus the other groups. ^f^. Not significant, versus the other groups.

**Figure 5:**
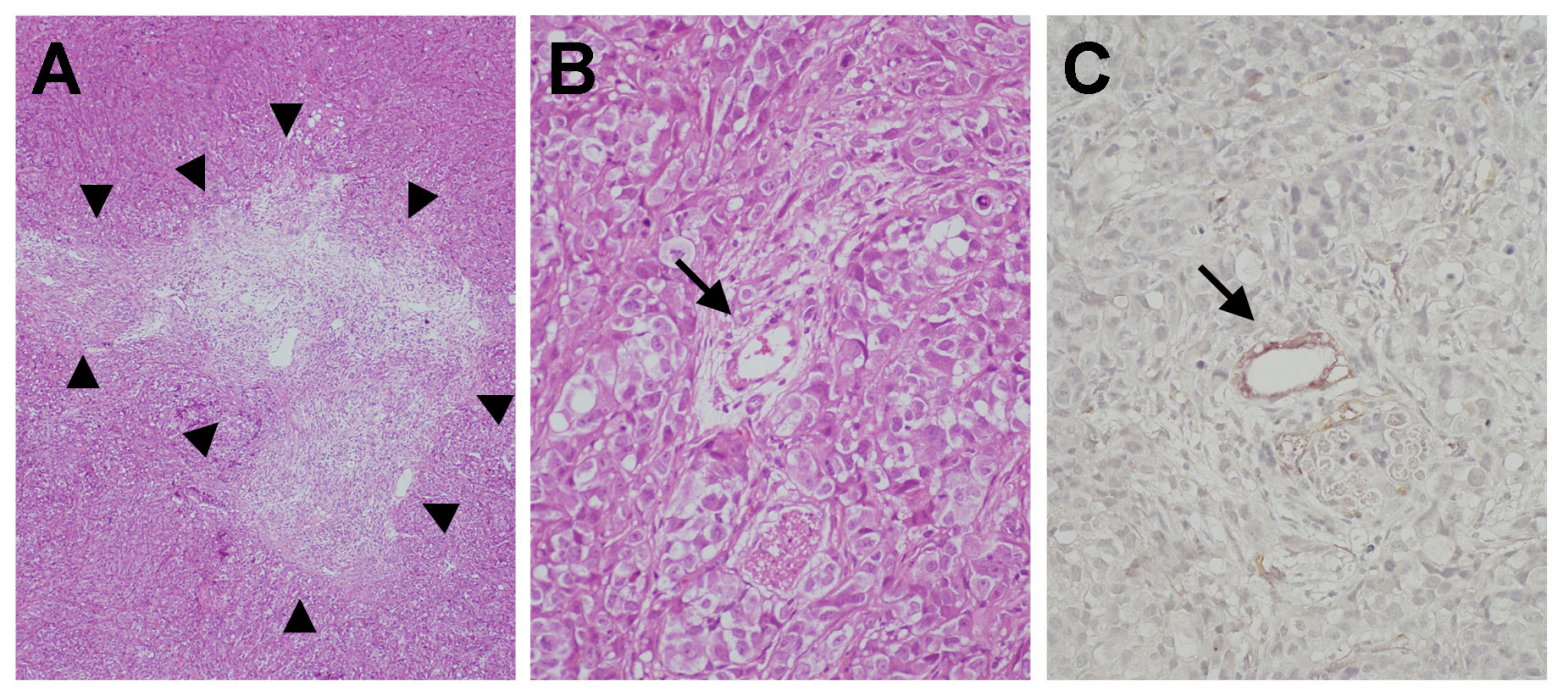
Photomicrograph of resected HAK-1B tumor. (A) Tumor cells are replaced with large granulation tissue at the middle of resected tumor. (arrowheads, HE staining, X20). (B) Artery-like blood vessels in the tumor (arrow, HE staining, X200). (C) Artery-like blood vessel in the tumor (arrow, CD34/α-SMA double-immunostain, X200).

The resected tumor of the PEG-IFN-α2a group showed granulation tissue at the middle of the tumor to various degrees (Figure 5). Arteries that appeared in the granulation tissue were excluded in blood vessel count within tumor. There was no significant difference in the number of blood vessels per unit area within the HAK-1B tumor and the expression of bFGF and IL-8 in the tumors between the PEG-IFN-α2a group and the Control group (Figure 5; Table 5). 

**Table 5:** Numbers of artery-like blood vessels, and Enzyme-linked immunosorbent assay (ELISA) of angiogenesis factors in human HCC tumors subcutaneously transplanted in nude mice.

	Artery-like blood vessel^b^ (Number of vessels/mm2)	Levels in the tumor lysate^c^ (pg/40 μg cellular protein)	
Treatment group^a^	Inside of tumor	bFGF	IL-8
Control	0.104 ± 0.02^d,e^	14.0 ± 1.8^e^	2.8 ± 1.0^e^
PEG-IFN-α2a (0.06 μg)	0.194 ± 0.05	19.8 ± 2.1	4.9 ± 1.3

^a^ Cultured HAK-1B cells (1.0 X 10^7^) were subcutaneously transplanted into nude mice. Five groups of 8 mice received either phosphate-buffered saline (PBS) (Control) or PBS with the different dosages of PEG-IFN-α2a (0.06–60 μg) once a week. Tumors of mice that received 0.6, 6 or 60 μg of PEG-IFN-α2a could not be used because the tumors were too small to evaluate. All mice were killed on the 15th day. ^b^ The number of artery-like blood vessels within tumor was counted on each section, and the average number per area in each group was obtained. ^c^ The expression levels of basic fibroblast growth factor (bFGF) and IL-8 of the resected tumors were measured by ELISA. ^d^ Mean ± SE. ^e^. Not significant, versus the PEG-IFN-α2a (0.06 μg) group.

## Discussion

In the *in vitro* study, we showed that PEG-IFN-α2a inhibit the growth of 8 and 11 out of 13 cell lines in a time- and dose-dependent manner, however, PEG-IFN-α2a was apparently less active on an IC50 basis, compared with either PEG-IFN-α2b or IFN-α2b or consensus IFN-α or BALL-1 lymphoblastoid IFN-α which was tested in the same experimental condition in our previous reports [10,18,21]. For example, IC50 for HAK-1B cells was approximately 253 ng/ml of PEG-IFN-α2a, 13.1 ng/ml of PEG-IFN-α2b, 2.4 ng/ml of IFN-α2b, 0.7 ng/ml of consensus IFN-α and 1.1 ng/ml of BALL-1 lymphoblastoid IFN-α. On the other hand, in the *in vivo* study, s.c. injection of PEG-IFN-α2a once a week showed better antitumor effect on a tumor volume or weight basis, compared with that of non-pegylated IFN-α2a. These results might support our hypothesis that continuous contact with IFNs induces strong *in vivo* antitumor effects, and are not surprising because it was reported that PEG-IFN-α2a showed less active in vitro antiviral activity and but had much more *in vivo* antitumor activity than non-pegylated IFN-α2a [23]. We also showed that PEG-IFN-α2a can inhibit the proliferation of CHC cell lines as well as HCC. In MTT assay, the growth of KMCH-1 was well suppressed although another CHC cell line, KMCH-2 was not. One possible explanation for the different sensitivity between KMCH-1 and KMCH-2 is that the origin of KMCH-1 is CHC, classical type and that of KMCH-2 is CHC with stem-cell features, intermediate-cell subtype according to the latest WHO classification [24]. Such a stem-cell properties of the tumor might be the reason for IFN resistance. Another interesting finding in the *in vitro* study is the discrepancy between the results of MTT assay and apoptosis detection assay. When HAK-1B or KIM-1 was cultured with PEG-IFN-α2a, IC50 for HAK-1B was much lower than that for KIM-1 although HAK-1B showed lower rate of apoptotic cells than KIM-1. These findings suggest that there might be some mechanisms other than apoptosis, which affect the sensitivity to antitumor effects of PEG-IFN-α2a. We previously reported that both pegylated and non-pegylated IFN-α inhibited the proliferation of cultured HCC cells by inducing the cell-cycle arrest [10,18]. The expression of interferon receptor on tumor cells might be a possible factor related to antitumor effect. For instance, Nagano et al reported that the expression of this type I IFN receptor on HCC tissue might be a useful predictor to find potential responder to INF-α/5-fluorouracil combination therapy [25]. Immunomodulation by IFNs has also been well studied as a factor related to antitumor effect. In this study, we used athymic mice, which lack mature T-cell, and human IFNs. Since IFNs are species-specific [26], we surmise that this immunomodulatory effect is limited in our study, but this should be confirmed in the future study using mouse IFN. 

 Morphological observation of the subcutaneous tumors of nude mice revealed that s.c. injection of PEG-IFN-α2a induce the significant increase of apoptotic cells compared with Control group. This result in the *in vivo* study is consistent with that in the *in vitro* study showing characteristic changes of apoptosis after adding PEG-IFN-α2a. Although the inhibition of angiogenesis as well as the induction of apoptosis is regarded as one of the biological effects of IFNs, there was no significant difference in the number of artery-like blood vessels of the subcutaneous tumors between the control and treatment groups. There are two possible explanations of this finding. Firstly, PEG-IFN-α2a was less effective for mouse endothelial cells compared with human cancer cells due to the species specificity of human IFNs. Secondly, it might be difficult to visualize the alteration in the number of vessels in order to examine the efficacy of drugs that possess antiangiogenic activity. Hlatky et al explained in their review article that the reason is that the tightness of the coupling between vessel drop-out and tumor-cell drop-out after the treatment is different [27]. We had observed similar findings in our previous report in which human HCC tumors subcutaneously transplanted in nude mice showed much apoptosis in either PEG-IFN-α2b or IFN-α2b treatment group compared with the Control group, but no significant difference in the number of blood vessels [10]. Kojiro et al also showed that s.c. injection of BALL-1 lymphoblastoid IFN-α increase the number of artery-like blood vessels and the protein expression of bFGF within HCC xenograft tumors in spite of the significant decrease of actual tumor weight [28]. In contrast, Dinney et al showed that IFN-α2a decreases the blood vessel density and the expression of bFGF in orthotopic xenograft model of bladder tumor [29]. The reason for these contrary ﬁndings remains unclear and further evaluation with caution is needed by using different doses and types of IFNs and different cell lines, not only in subcutaneous tumor model but also in orthotopic model. 

 The association between IFN therapy and occurrence or recurrence of HCC has been investigated in some reports. HALT-C trial group showed in their randomized control trial in a large cohort that long-term PEG-IFN-α2a therapy does not reduce the incidence of HCC among patients with chronic HCV infection who have previously failed to achieve a sustained virologic response to therapy [30]. Among only patients with cirrhosis, long-term PEG-IFN-α2a therapy reduced a risk of HCC after a long-time observation [31]. EPIC study group also showed long-term PEG-IFN-α2b therapy does not prevent HCC [32]. On the other hand, Nishiguchi et al reported that long-term IFN-α therapy after curative resection of HCV-related HCC prolongs the survival rate, although preventive effect of intrahepatic recurrence was marginal [33]. Sakaguchi et al also showed that among patients who underwent radical radiofrequency therapy for HCV-related HCC, long-term IFN-α2b therapy reduced the recurrent rate of HCC [4]. These reports with conflicting results may be suggesting that IFN therapy is effective only after the initial curative treatment of HCV-related HCC. In addition, there are several reports that support that IFN therapy prevents the development of HCC among patients with chronic HBV infection or those underwent curative resection of HBV-related HCC [5,7]. Thus the chemopreventive effect of IFNs against HCC are still controversial, and mechanisms behind that remain unclear. Antiviral effect against HBV and HCV, which are risk factors for HCC, and immunomodulatory effect of IFNs are regarded as main mechanisms. Another possible mechanism is that IFNs may suppress the growth of clinically undetectable HCC due to their direct antitumor effect. Our finding in the current study provide the evidence that PEG-IFN-α2a possesses the direct antitumor effect against HCC.

 In conclusion, we demonstrated antitumor effect of PEG-IFN-α2a for human liver cancer cells *in vitro* and *in vivo* and our results suggest that longer contact to IFNs may induce stronger antitumor effect in body. PEG-IFN-α2a might be a possible treatment option for HCC as well as chronic viral hepatitis. Further studies are needed from both molecular and clinical view points in order to find out particular patient group those respond to this therapy. 
